# Alert for Imported Malaria in Non-Endemic Areas: A Case Report of Atypical Falciparum Malaria in a Young Child and Diagnostic Experience

**DOI:** 10.3390/tropicalmed11010015

**Published:** 2026-01-06

**Authors:** Jiali Feng, Yang Zhou, Bo Zhang, Ming Huang

**Affiliations:** Department of Laboratory Medicine, Tongji Hospital, Tongji Medical College, Huazhong University of Science and Technology, Wuhan 430030, China

**Keywords:** imported malaria, *Plasmodium falciparum*, pediatric malaria, atypical presentation, diarrhea, laboratory diagnosis

## Abstract

Background: Although China has eliminated indigenous malaria, imported cases, particularly among young and middle-aged workers returning from Africa, constitute a major challenge for current epidemic prevention and control. In contrast, imported malaria in children is extremely rare and often subject to diagnostic delays in non-endemic areas due to atypical clinical presentations. Case presentation: We report a case of a 2-year-11-month-old boy who returned from Sudan, a malaria-endemic region, presenting with fever and diarrhea as the initial symptoms. The case was identified by the laboratory through the blood routine re-examination rules, crucially informed by the patient’s epidemiological history. The diagnosis was ultimately confirmed as *Plasmodium falciparum* malaria by rapid diagnostic testing and microscopic examination. Conclusion: This diagnostic pathway exemplifies a closed-loop model of “clinical suspicion → targeted laboratory testing → definitive pathogen identification.” It provides a practical framework for the early detection and diagnosis of pediatric imported malaria with atypical presentations in non-endemic areas.

## 1. Introduction

Malaria, a deadly vector-borne infectious disease, remains a pressing global public health concern, with the highest burden in Africa [1]. Children under five years of age bear the highest mortality, accounting for 76% of all malaria deaths on the continent [2]. Although China achieved elimination of indigenous malaria in 2017 [3], the increasing international travel has rendered imported malaria the predominant form of the disease. The number of reported imported cases in China has shown an upward trend in recent years, with the majority occurring in young and middle-aged men who have worked in Africa [4,5]. In sharp contrast, imported malaria among children under 14 years, especially infants under three, is exceedingly rare. They constituted only 0.09% of reported cases from 2017 to 2020 [6]. The youngest case reported in Wuhan between 2010 and 2023 was 18 years old [7], which means no cases under the age of 18 were reported in that area during that period.

However, once infected, children face a much higher risk of severe illness and mortality compared to adults. In malaria-endemic areas of Africa, children under five represent the population at the highest risk for morbidity and death [8]. In non-endemic regions, diagnostic delay remains a key risk factor for poor prognosis in imported cases [4,9]. Studies indicate that a delay exceeding three days from symptom onset to diagnosis significantly increases the risk of severe malaria, and an interval of 4–12 days is considered a definite risk factor [10,11]. Therefore, early diagnosis is paramount for effective management and treatment of malaria in non-endemic areas.

The clinical manifestations of malaria are complex and non-specific. Classic symptoms—intermittent chills, high fever, and sweating caused by the periodic schizogony of *Plasmodium* in red blood cells [12]—often overlap with those of common illnesses such as influenza and sepsis. Consequently, diagnosis based solely on clinical presentation is highly unreliable, especially in children [8,13,14]. Among the various atypical presentations, gastrointestinal symptoms constitute a major diagnostic pitfall. Studies show that nausea, vomiting, abdominal pain, and diarrhea are frequent in pediatric malaria [15]. Cases dominated by these symptoms are often misdiagnosed as common childhood conditions like acute gastroenteritis, leading to delays in diagnosis [14]. Therefore, in low-endemic settings, high clinical suspicion is essential for children with a travel history to endemic areas, particularly when they present with atypical symptoms.

This article reports a case of imported *Plasmodium falciparum* (*P. falciparum*) malaria in a young child, presenting with fever and acute diarrhea as the primary symptoms. This is potentially the youngest such case reported in Wuhan, China, since 2010. The prompt diagnosis was facilitated by the clinical laboratory’s strict adherence to blood film re-examination protocols and the accurate reconstruction of the case’s epidemiological history. This case highlights the critical need for vigilance regarding atypical pediatric malaria in non-endemic settings. It also underscores the importance of clinical laboratories fully utilizing clinical data, enhancing morphological diagnostic skills for *Plasmodium*, and leveraging re-examination rules and instrument flags. These measures can provide vital clues for the timely diagnosis of atypical malaria.

## 2. Case Presentation

The patient, a 2-year-11-month-old boy, had entered China from Sudan on 1 September 2025. He presented at the pediatric emergency department on 10 September 2025, with a 5-day history of fever and diarrhea. His recorded peak temperature was 39.6 °C, exhibiting an irregular fever pattern without chills. He experienced several episodes of watery diarrhea daily, accompanied by mild cough, nasal congestion, and rhinorrhea, but no vomiting. Physical examination revealed a conscious and responsive child with stable breathing. Findings were notable only for pharyngeal congestion and grade I tonsillar hypertrophy. Lung auscultation revealed coarse breath sounds without rales or wheezing. Cardiac and abdominal examinations were unremarkable. The patient had a known history of iron-deficiency anemia.

Initial laboratory tests in the emergency department showed a slight increase in neutrophil percentage, decreased hemoglobin (Hb) and hematocrit (HCT), and increased red cell distribution width (RDW), consistent with microcytic heterogeneous anemia. C-reactive protein (CRP) was significantly elevated. Based on these findings, the initial diagnosis was gastroenteritis complicated by a respiratory tract infection. Treatment commenced with ibuprofen, cefuroxime, probiotics, and ribavirin.

**Figure 1:**
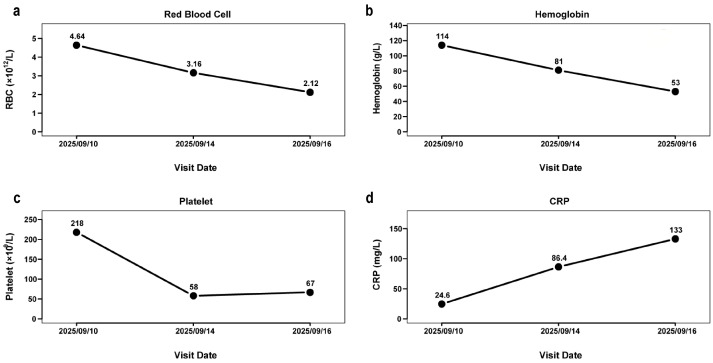
Temporal trends of red blood cell count, hemoglobin, platelet count, and reactive protein in the patient during the outpatient course. (**a**) Trend of red blood cell count over time during the patient’s outpatient visits. The patient’s RBC count decreased rapidly within a short period. (**b**) Trend of hemoglobin levels over time during the patient’s outpatient visits. The patient’s hemoglobin level decreased rapidly within a short period. (**c**) Trend of platelet count over time during the patient’s outpatient visits. The patient’s platelet count decreased rapidly within a short period. (**d**) Trend of C-reactive protein (CRP) levels over time during the patient’s outpatient visits. The patient’s CRP level increased rapidly within a short period.

On 14 September 2025, the child returned to the clinic due to persistent high fever and cough, although the diarrhea had slightly improved. Re-examination confirmed persistent coarse breath sounds in both lungs, and hospitalization was recommended. The family declined admission and instead chose outpatient intravenous ceftriaxone treatment. On 16 September 2025, the patient’s condition worsened, prompting repeat complete blood count (CBC), CRP, and chest imaging. The CBC results demonstrated a marked decline in red blood cell count, hemoglobin, and platelet count compared to the previous examination, indicating progressively worsening anemia and severe thrombocytopenia, alongside further elevation of CRP (Figure 1). These significant abnormalities triggered the laboratory’s predefined re-examination rules.

**Figure 2:**
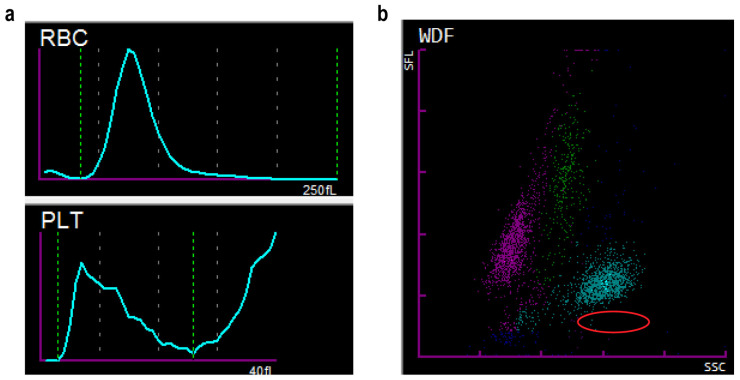
Scatter plots and histograms from the Sysmex XN hematology analyzer. (**a**) Histograms from the Sysmex XN hematology analyzer. The platelet histogram reveals the presence of an irregular serrated tail. (**b**) White blood cell scatter plot from the Sysmex XN hematology analyzer’s WDF channel. The area marked by a red circle indicates red blood cells infected with malaria parasites; no such cell population was detected in this patient’s sample.

**Figure 3:**
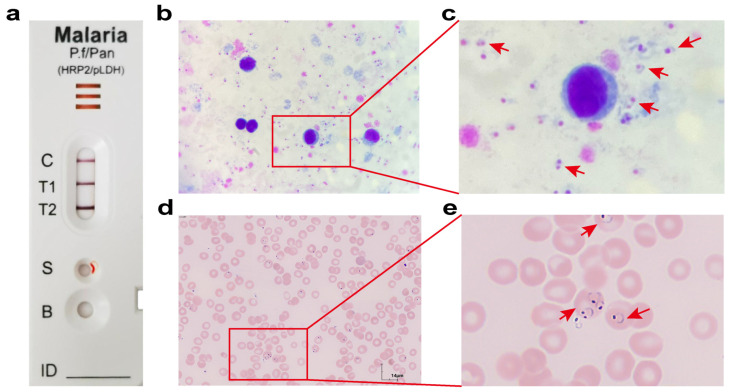
Laboratory findings from the patient’s blood sample. (**a**) Result of the malaria RDT. (**b**) A large number of *Plasmodium* ring forms are observed in the Giemsa-stained thick blood smear. (1000× magnification) (**c**) Higher-magnification view of the area boxed in red in (**b**), revealing ring-form trophozoites (red arrow) in the thick blood smear. (**d**) The Wright-Giemsa-stained thin blood smear reveals infected erythrocytes containing multiple ring forms. (Scale bar: 14 μm) (**e**) Higher-magnification view of the area boxed in red in (**d**), revealing ring-form trophozoites (red arrow) in the thin blood smear.

Laboratory personnel, noting the pronounced changes, re-examined the blood sample. The hematology analyzer flagged platelet aggregation, and the platelet histogram displayed an irregular saw-toothed tail (Figure 2a). A blood smear was immediately prepared for manual microscopy. Given the patient’s progressive cytopenias and the critical epidemiological history of recent return from a malaria-endemic area, malaria infection was strongly suspected. While manually reviewing the smear for platelet verification, a thick blood film was prepared for malaria morphology testing, and the patient’s white blood cell scatter plot was re-evaluated. The WNR channel of the Sysmex XN series hematology analyzer, which exploits the inability of malaria parasites to be lysed by hemolytic agents, showed a cluster of purple-red cells below the neutrophil population, triggering an iRBC alarm. Notably, the patient’s white blood cell scatter plot lacked the typical cell cluster pattern usually associated with *Plasmodium* infection and the specific iRBC alarm (Figure 2b). Despite this, given the high clinical suspicion, the laboratory performed a gold-labeled *Plasmodium* rapid diagnostic test (RDT). The RDT used in our laboratory was the BioPerfectus Malaria Antigen *P. falciparum/Pan-Plasmodium* Test (Catalog Number: SC10110), which targets the histidine-rich protein-2 (HRP-2) antigen specific to *P. falciparum* and the pan-plasmodium lactate dehydrogenase (pan-pLDH) antigen common to other malaria species (*Plasmodium vivax*, *Plasmodium ovale*, and *Plasmodium malariae*). Interestingly, the patient’s RDT showed two positive bands. According to the interpretation criteria provided in the manufacturer’s instructions, this result indicates a *P. falciparum* infection, and does not rule out the possibility of a mixed infection (Figure 3a).

Subsequent microscopic examination of the thick blood film revealed numerous *Plasmodium* ring forms (Figure 3b,c). On the thin blood film, the ring forms appeared small and delicate, resembling a bird in flight, with the ring occupying approximately one-fifth of the red blood cell diameter. Most rings contained two chromatin dots, with minimal cytoplasm. No gametocytes or other developmental stages were observed. The infected red blood cells maintained normal morphology, with the majority harboring 2–3 parasites (Figure 3d,e). These morphological features were consistent with *P. falciparum* malaria infection. The laboratory promptly communicated these critical findings to the physician. The patient’s blood sample was sent to the Chinese Center for Disease Control and Prevention (CDC) for confirmatory testing, where PCR-based molecular diagnostic methods were used to identify the *Plasmodium* species. The PCR detection kit used was the *Plasmodium Fluorescence* PCR Detection Kit manufactured by Jiangsu Hechuang Biotechnology Co., Ltd. (Catalog Number: CN31-1, Zhenjiang, China). This kit employs a real-time fluorescence quantitative PCR method capable of detecting *Plasmodium genus*, *P. falciparum*, *Plasmodium vivax*, *Plasmodium ovale*, and *Plasmodium malariae.* Confirmatory molecular detection performed by the China CDC identified *P. falciparum* as the etiological agent, consistent with the national standard WS/T 10030—2025 Detection of *Plasmodium* spp.—Nucleic Acid Identification of the Species. [16]. The patient was transferred to a specialized infectious disease hospital, where they received standardized antimalarial treatment with an Artemisinin-based Combination Therapy (ACT) regimen. The patient recovered and was discharged after a 10-day hospitalization.

## 3. Discussion

The rise in international travel poses a continuing challenge to malaria control in China, which, despite its WHO-certified malaria-free status, now faces imported cases as the primary concern [17,18]. Consequently, the early identification and diagnosis of these cases are critical for mitigating the risk of importation and potential local re-establishment of the disease, particularly in patients with atypical symptoms. This report describes a case of imported *P. falciparum* malaria in a young child from Sudan, likely the youngest reported in Wuhan in over a decade. The patient’s presentation, dominated by fever and acute diarrhea, underscores the critical lessons for recognizing such rare and atypical cases in low-endemic settings.

The successful diagnosis of this case primarily resulted from a high index of suspicion regarding the epidemiological history. Compared with adults, pediatric malaria presents with notably more non-specific clinical manifestations. Classic symptoms such as chills and arthralgia are less common, while high fever, somnolence, and gastrointestinal symptoms (e.g., nausea, vomiting, diarrhea) predominate [12,15]. The classic periodic fever pattern—occurring every 48 or 72 h—is present in fewer than a quarter of pediatric cases. Conversely, children are more susceptible to high fevers exceeding 40 °C and febrile convulsions [4]. The irregular, intermittent fever pattern observed in this case further complicated the diagnosis. Here, the absence of paroxysmal chills, combined with diarrhea and a history of iron-deficiency anemia, could easily have led to an initial misdiagnosis of acute gastroenteritis or an anemia-related disorder, representing a significant diagnostic pitfall. However, the key epidemiological clue—the onset of symptoms within 9 days of arrival from a high-endemic area (Sudan)—proved to be the most compelling indicator of imported malaria. This underscores the critical importance of systematically obtaining a travel history during triage and initial clinical assessment.

The complete blood count (CBC) revealed a progressive decline compared to previous results: the red blood cell (RBC) count decreased to 2.12 × 10^12^/L, hemoglobin (Hb) to 53 g/L, and platelets to 67 × 10^9^/L. Simultaneously, the C-reactive protein (CRP) level rose further to 133 mg/L. The progressive anemia is primarily attributable to the destruction of *Plasmodium*-infected erythrocytes and enhanced splenic clearance of parasitized red blood cells. Severe thrombocytopenia, a hallmark of malaria, results from peripheral consumption, immune-mediated destruction, and potential bone marrow suppression. The sustained systemic inflammatory response triggered by the malaria infection is the principal cause of the marked elevation in CRP. In summary, these findings indicate that the patient’s condition was progressively worsening. Within the laboratory, the dramatic CBC changes, particularly the sharp decline in platelets, activated the re-examination protocols, demonstrating the pivotal role of standardized laboratory procedures in flagging potential infections. The implementation of these re-examination rules was instrumental in the prompt identification of this atypical malaria case. Literature consistently reports thrombocytopenia as a hallmark feature of malaria, occurring in approximately 45% to 71% of imported cases in both adults and children [15,19]. The platelet aggregation alarms and abnormal histograms served as the initial critical triggers for deeper laboratory investigation. This highlights the effectiveness of structured re-examination rules and the indispensable role of laboratory technologists’ expertise in identifying potential rare cases.

Notably, the Sysmex XN-series hematology analyzer in this case did not flag any typical malaria-specific alarms, such as an abnormal white cell differential scattergram or a positive ‘iRBC’ flag. This may be attributed to low parasitemia and the absence of gametocytes in the peripheral blood. The Sysmex XN analyzer primarily triggers the iRBC alarm by detecting particulate intra-erythrocytic material of sufficient size and density, such as parasite nucleic acid and hemozoin pigment. The early ring forms of *P. falciparum* are particularly small, occupying a minimal volume of the red blood cell, and their relatively low nucleic acid content makes them less likely to be detected [20]. Therefore, negative automated analyzer results cannot reliably exclude a malaria diagnosis, and definitive identification of malaria parasites on blood smear microscopy remains the gold standard.

The confirmation of the pathogen in this instance illustrated an effective multi-modal diagnostic workflow. The RDT offered a rapid and critical preliminary result, effectively bridging the gap between clinical suspicion and initial pathological evidence. The observed two-band pattern was consistent with a *P. falciparum* infection, although a mixed-species infection could not be definitively ruled out. This RDT format detects *P. falciparum*-specific HRP2 and genus-wide pan-pLDH. It is documented that *P. falciparum* expresses pLDH, which can lead to a dual-positive test result even in the absence of a co-infection [21]. However, as a serological method, RDTs are susceptible to false positives and cross-reactivity. Consequently, they must be considered a preliminary screening tool, with definitive diagnosis requiring microscopic visualization of the parasite and, where accessible, confirmation by PCR [8].

Microscopic examination played a vital role in species identification. *P. falciparum* possesses distinctive morphological characteristics that differentiate it from other *Plasmodium* species. Due to the sequestration property of *P. falciparum*, red blood cells containing mature trophozoites and schizonts adhere to deep tissue capillaries (e.g., in the brain and heart). Consequently, peripheral blood smears typically reveal only ring forms, while developing trophozoites, schizonts, and often gametocytes are absent [22,23]. The presence of “rings only” is the most characteristic peripheral blood finding in *P. falciparum* infection. The morphology of the parasites and infected red cells in the patient’s thin film aligned with typical *P. falciparum* infection, and the absence of other parasite stages effectively ruled out mixed infection. PCR results from the Chinese CDC later confirmed the morphological findings.

Finally, the atypical gastrointestinal symptoms in this case warrant further discussion. Although the co-occurrence of malaria and diarrhea is well-documented, with reported incidence rates ranging from approximately 1.6% to 44% [24], the underlying causal relationship is not fully elucidated. Some studies suggest that following *Plasmodium* infection, parasitized red blood cells adhere to and sequester within the microvascular endothelium of organs such as the intestines, leading to extensive microcirculatory obstruction [25]. This obstruction, combined with ischemic injury, disrupts intestinal barrier function and increases permeability [26], potentially inducing inflammatory exudation and thereby causing secretory or exudative diarrhea. Consequently, diarrhea appears to be a direct outcome of malaria infection. Conversely, other research indicates that the dual impact of impaired intestinal barrier function and microbial dysbiosis heightens patients’ susceptibility to enteric pathogens [27]. Thus, the observed diarrhea may result from a co-infection involving both *Plasmodium* and an intestinal pathogen, rather than from *Plasmodium* alone [26,27,28]. The resolution of diarrhea with antibiotic therapy lends indirect support to the co-infection hypothesis, implicating an enteric pathogen alongside *Plasmodium*, rather than *Plasmodium* itself as the sole cause. Regrettably, the patient did not provide a stool specimen during the clinical visit, which precluded definitive identification of an intestinal pathogen and left the true etiology of the diarrhea undetermined. This phenomenon underscores a key clinical implication: for febrile children with a history of travel to endemic areas who present with diarrhea, clinicians should consider not only malaria but also maintain a high index of suspicion for concurrent bacterial gastroenteritis and initiate relevant diagnostic investigations promptly.

This study has several limitations. First, due to the referral policy for malaria cases, the patient was transferred to a specialized infectious disease hospital for further treatment, and we were unable to obtain subsequent therapeutic and follow-up data. Although attempts were made to contact the patient to acquire this information, we were unsuccessful, likely because the patient had left China. While the absence of these data affects the completeness of this case report, it does not compromise the core conclusions of the study, which focus on clinical recognition and diagnostic reasoning regarding imported malaria in children in low-prevalence areas. Second, during the patient’s clinical management, stool examination was not promptly recommended to identify the causative pathogen of diarrhea. Therefore, the hypothesis that diarrhea resulted from co-infection with malaria and an intestinal pathogen could only be indirectly supported by the resolution of symptoms following antibiotic therapy. Finally, in the diagnostic workflow for suspected malaria, rapid diagnostic tests (RDTs) should be employed as the initial screening tool rather than being performed after microscopic examination. In future clinical practice, adherence to standardized malaria diagnosis and treatment protocols will be emphasized to further improve the quality of care.

## 4. Conclusions

In summary, the diagnosis of this case was facilitated by a sequential diagnostic pathway: clinical suspicion → laboratory re-examination → pathogen confirmation. This experience underscores that in low-endemic settings, obtaining a thorough travel history is the cornerstone of accurate diagnosis for febrile children with atypical presentations. Concurrently, clinical laboratories must adhere to re-examination protocols, recognize the limitations of automated analyzers, maintain microscopy as the diagnostic gold standard, and integrate multiple techniques to ensure the timely diagnosis of atypical malaria.

## Data Availability

The original contributions presented in this study are included in the article. Further inquiries can be directed to the corresponding authors.
